# Benefits and Effects of Laser-Based Techniques in Complementary Maxillary Expansion: A Narrative Review

**DOI:** 10.7759/cureus.74500

**Published:** 2024-11-26

**Authors:** Theoklitos Tsaprazlis, Dimitrios Filippou, Maria Mavragani

**Affiliations:** 1 Department of Anatomy, National and Kapodistrian University of Athens, Athens, GRC; 2 Department of Orthodontics, University of Bergen, Bergen, NOR

**Keywords:** laser therapy, maxillary expansion, orthodontics, palatal expansion technique, photobiomodulation

## Abstract

Transversal maxillary deficiency is a prevalent skeletal issue that can be addressed using various devices and methods, including rapid maxillary expansion (RME) and surgically assisted rapid maxillary expansion (SARME). These techniques involve the separation and regeneration of the midpalatal suture (MPS). Laser therapies, such as low-level laser therapy (LLLT) and photobiomodulation (PBM), have been proposed to improve biological wound or bone healing. The research sought to systematically evaluate the evidence regarding the advantages and effects of laser-based techniques in conjunction with RME or SARME.

An electronic search was conducted through September 2024 in the PubMed database utilizing relevant Medical Subject Headings, without any time constraints. A total of 78 publications were identified with the keywords ‘’Orthodontics’’, ‘’Laser Therapy’’, ‘’Palatal Expansion Technique’’, ‘’Maxillary Expansion’’ and ‘’Photobiomodulation’’. Only 17 were included in this research (11 were clinical trials, and six were systematic reviews). In addition, a secondary manual literature search was performed from the references of included articles and existing systematic reviews, adding one extra article.

Despite the varying intervention protocols used in each study, they consistently demonstrated that laser techniques can improve bone regeneration after RME or SARME procedures. Although the available evidence is somewhat sparse, these methods seem to offer a promising solution for alleviating pain during MPS expansion. To establish definitive outcomes and develop a reliable clinical protocol, longer randomized clinical trials are essential to ensure the practicality of this technique.

## Introduction and background

A posterior crossbite is a prevalent malocclusion found in both kids and adults. It can occur bilaterally or unilaterally, with unilateral cases further divided into functional or true variations. Maxillary constriction can lead to various complications, including aesthetic concerns, occlusal misalignment, a narrowed pharyngeal airway, increased nasal resistance, mouth breathing habits, altered tongue posture, and obstructive sleep apnea. Among malocclusions, transverse discrepancies are the most commonly observed issues. Three primary approaches are suggested for addressing maxillary constriction: non-surgical orthodontic rapid maxillary expansion (RME), surgically assisted rapid palatal expansion (SARPE), and segmental LeFort osteotomy. RME has gained traction owing to its multiple benefits on overall health and its success rate in correcting dental or skeletal transverse maxillary discrepancies. Furthermore, RME can improve nasal breathing by widening the nasopharyngeal airway [1]. In this context, RME generates substantial forces transmitted to the midpalatal suture (MPS) through the teeth, leading to skeletal changes [2]. In cases of significant transverse discrepancies (>7 mm), limited intercuspid widths, and deficits in maxillary arch length coupled with crowding, SARPE is recognized as the preferred treatment. The clinical approach to maxillary expansion consists of an active phase characterized by induced lateral forces, succeeded by a passive phase involving retainer maintenance. A notable challenge of RME is the swift relapse of results in the absence of retainers. One factor contributing to treatment relapse is inadequate bone regeneration at the MPS. As a result, researchers are focusing on methods to enhance bone regeneration within the MPS [1].

In dental practices, low-level laser therapy (LLLT) and high-intensity laser therapy (HILT) are mainly employed for surgical procedures and biostimulation treatments. LLLT is a non-invasive method that administers low energy without raising the temperature at the treatment site above normal ranges. This economical approach is adaptable for numerous orthodontic purposes, as it initiates temporary biochemical changes that lead to important biological effects. LLLT has shown beneficial effects on cytokine expression, wound healing, angiogenesis, cell proliferation, and the repair and remodeling of bone through enhanced collagen production. High-frequency pulsed low-level diode laser treatment improves the healing of tooth extraction sites. After alveolar bone healing, LLLT can promote a more uniform trabecular structure. In vivo research underscores the advantages of LLLT during maxillary expansion, facilitating improved bone formation and osteoclast differentiation. A recent extensive textbook on dental laser applications features a chapter dedicated to orthodontic interventions, discussing techniques such as gingivoplasty, exposure of impacted teeth, bracket placement, pain relief, and tooth mobility. Nonetheless, it falls short of specifically examining LLLT’s effects on RME and the subsequent success of bone regeneration. [1,3,4].

The effects of laser therapy on bone regeneration after MPS opening are still unclear due to a lack of conclusive evidence. This ambiguity may arise from the lack of a systematic approach to gathering pertinent data, which could impede ongoing research efforts [5]. Therefore, this review intends to gather and rigorously assess data from available studies in this area to ascertain whether laser therapy positively influences bone healing following RME.

## Review

Materials and methods

**Figure 1:**
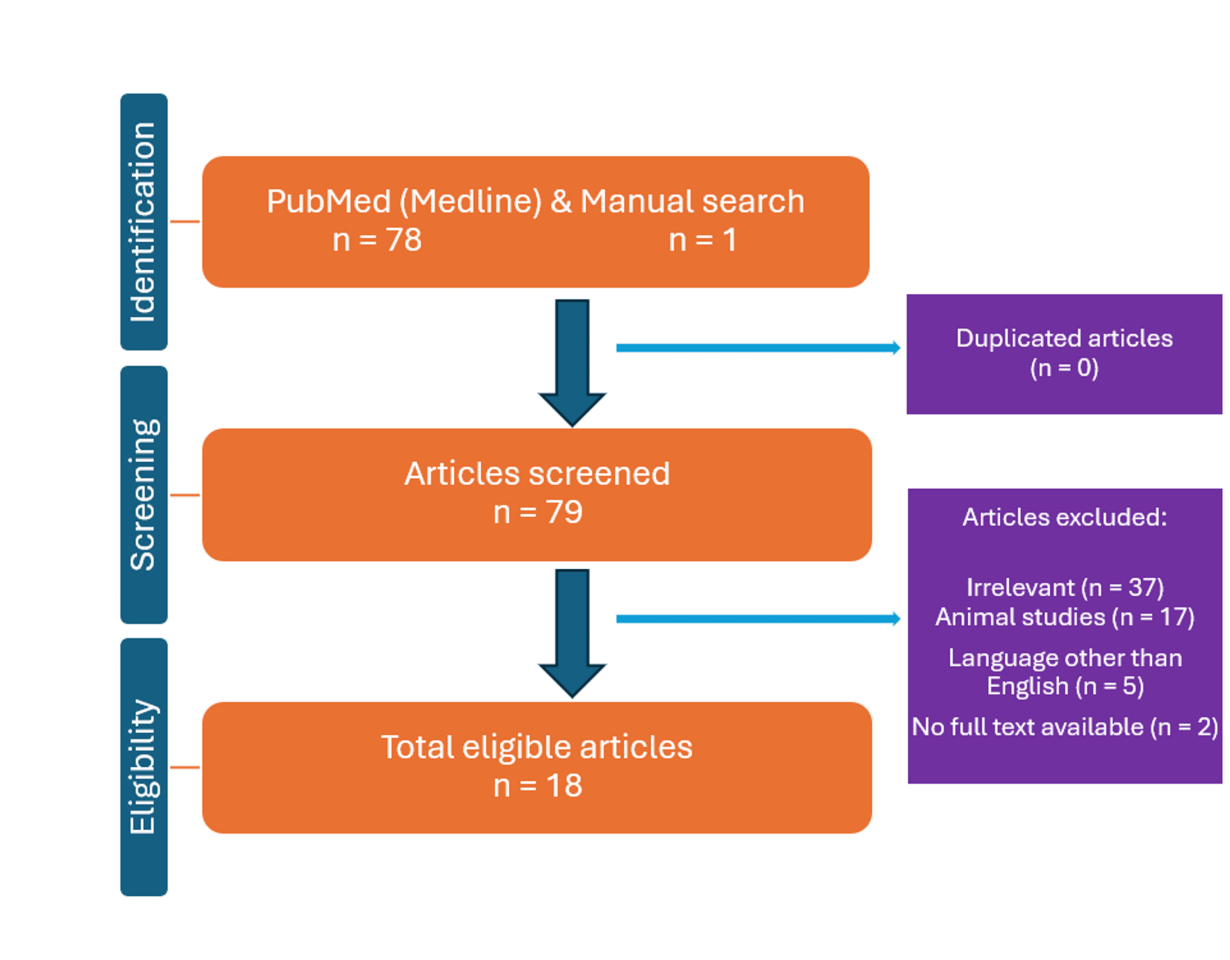
Study flowchart diagram of the included studies.

A systematic search was conducted in the PubMed database to perform our study, with no restriction on the year of publication. The Medical Subheading (MeSH) terms related to Orthodontics, Palatal Expansion Technique, and Laser Therapy, as well as the search strategy built for the aforementioned databases are depicted in Figure 1. Free text words, such as “orthodontic*”, “Palatal Expansion”, “Maxillary Expansion”, “Laser” and ‘’Photobiomodulation’’ were also used during a second search in the Medline database. Given the limited evidence regarding the impact of laser therapy on midpalatal bone healing, the authors opted to include all relevant scientific papers in this review to enhance the evidence on the topic. The criteria for excluding articles from this review included studies not relevant to laser therapy, animal studies, manuscripts in languages other than English, duplicated publications, and finally, papers with no full text available.

Results

The initial search yielded 78 articles from the PubMed database. There were no restrictions on the year of publication for the included articles. Sixty-one articles were excluded based on specific criteria. Among these, 37 did not relate to the topic of this review, 17 were studies involving animals, five were not written in English or Greek, and the full text was inaccessible for the final two. A secondary manual literature search of the selected articles was conducted, resulting in the addition of one extra article. Consequently, this work was based on a total of 18 articles. Of these, 10 were clinical trials, seven were reviews, and one was a case report. Regarding focus, 14 papers explored the application of laser treatment, while three investigated photobiomodulation (PBM).

Discussion

The overall sample consisted of 351 participants. Each study included anywhere from one participant, as seen in a case report [6], to 82 participants [7]. The research focused on individuals aged between 6 and 54 years [2,6-15]. Among these studies, seven involved minors [2,7,8,10-12,14] and six involved adults [6,7,9,13-15]. In terms of orthodontic techniques for palate expansion, seven articles reported using RME [2,8,10-12,14,15], while five used SARPE [6,7,9,13,15]. Concerning the types of lasers employed in the studies, two utilized LED PBM [2,13], four employed Gallium Aluminum Arsenide (GaAlAs) [6,7,12,15], four used diode lasers [6,9-11], one used Indium Gallium Aluminum Phosphide (InGaAlP) [8], and one utilized Erbium:YAG [14]. In terms of laser wavelengths, two were at 660 nm [8,13], three were at 780 nm [7,11,12], one was at 824 nm [6], one was at 830 nm [15], one was at 850 nm [13], two were at 980 nm [9,10], and one varied from 450 to 835 nm [2]. The dosage applied varied between 1.5 J/cm² per point [6] and 140 J/cm² per point [15]. As for the entire duration of the laser application, it spanned from 2 minutes [14] to 2,240 seconds (around 37.3333 minutes) [7].

Safety

The safety of this proposed new therapeutic approach is of great importance and several studies have been focused on it. Following an analysis of the clinical studies regarding the safety of the applied technique, the subsequent findings were noted: Caccianiga et al. found no significant negative effects on tissues [2]. Garcia et al. reported that LLLT for biostimulation of bone does not negatively affect cell viability and likely enhances certain biological processes without inducing thermal responses. This includes promoting the proliferation and differentiation of osteoblasts that form nodules, as well as activating mitochondrial respiratory chain photoreceptors. These actions lead to changes in cellular ATP levels and stabilization of the cell membrane [8]. Moreover, Caccianiga et al. remarked that LLLT is a non-invasive technique. The Erbium:YAG laser is said to be less traumatic in comparison to methods such as piezosurgery or carbide bur [9]. Matos et al. noted that the study did not report any significant injuries; however, a few participants did experience minor gingival tissue damage around the bands [10]. De Rezende et al. corroborated Gasperini et al.'s claim that the GaAlAs laser is considered safe [7]. Cepera et al. pointed out that low-level laser usage is easy to manage, painless, and free from side effects [11]. Ferreira et al. emphasized that laser applications followed biosafety standards [12]. Angeletti et al. found that low-level lasers are characterized by the lack of detrimental thermal effects on the body, promoting cell biostimulation and thus improving regenerative capacity [15].

The examination of systematic reviews on safety indicated that Sant'Ana et al. emphasized the importance of diode lasers in high-intensity laser therapies for orthodontics, citing their superficial cutting ability, which improves safety by controlling penetration depth and reducing the chance of pulp injury [3]. Davoudi et al. reported that LLLT is advantageous for RME, as it does not produce side effects [1]. Skondra et al. further highlighted the absence of negative effects tied to LLLT [5]. Furthermore, Santinoni et al. verified that there were no harmful consequences [16]. Although a detailed search was performed to identify possible complications none of the studies referred to any minor or major, acute or long-term complication, suggesting the safety of the studied techniques.

Efficacy

In their clinical studies, Caccianiga et al. reported that pain levels at all evaluated time points and the maximum pain score were significantly lower in the experimental group (*P *< 0.05), with pain intensity decreasing more swiftly, approaching a level near zero within two to three days [2]. Garcia et al. observed that by day 75 following surgery, the irradiated cohort displayed a greater percentage of approximation in both the anterior (*P *= 0.008) and posterior (*P *= 0.001) upper sutures, but less approximation in the posterior superior suture (*P *= 0.040) when compared to the placebo group. A *P*-value of 0.05 was considered statistically relevant. Notably, the irradiated patients showed better approximation than those who were not irradiated (*P *= 0.056). In the group that underwent irradiation, 50% experienced notable approximation, while 30% achieved complete approximation, compared to 42% and 21% in the non-irradiated group, respectively (*P *= 0.450). This suggests that 27% of the irradiated patients had a greater degree of approximation at their maxillary suture margins compared to those who were not irradiated [8]. Caccianiga et al. found that all subjects reported successful correction of posterior unilateral crossbite while attaining functional occlusion. The average expansion observed was significantly greater on the affected side than on the unaffected side, with significant differences noted at the first premolars (*P *< 0.05), second premolars (*P *< 0.05), and first molars (*P *< 0.05) [9]. Meanwhile, Matos et al. noted that there was no significant difference in bone formation between the two groups (*P *= 0.2273). After three months, neither group had fully completed bone formation, nor were pain levels similar (*P *= 0.3940). However, pain was notably more severe during the initial seven days of treatment than on day 14 [10]. Research by de Rezende et al. found that the average increase in mouth opening was more pronounced in the laser group (GL) after bimaxillary orthognathic surgery. In females, the final average mouth opening was 27.86 mm (57.11%) for the control group versus 28.97 mm (61.85%) for the laser group. In males, the final mean values were 31.55 mm (55.31%) for controls compared to 36.98 mm (66.93%) for the laser therapy group, showing a significant difference (*P *= 0.00162) [7]. Cepera et al. found that their analysis of bone density revealed that laser treatment improved MPS opening and sped up the process of bone regeneration, utilizing a significance level of 5% (*P *< 0.05) across all evaluations [11]. Ferreira et al. demonstrated a statistically significant variation in optical density (OD) values when comparing T0 (post-disjunction) to T1 (after four months) within the laser-treated cohort (*P *= 0.00); however, no notable difference was observed in the control group (*P *= 0.20). Intergroup comparisons at T1 indicated a higher OD in the laser-treated group (*P *= 0.05), with the data analyzed using the paired Student’s t-test (α = 0.05%) [12]. Abreu et al. observed through radiographic evaluations that there was an advanced development of new bone within the median palatine suture. Radiopacity showed a decrease on both days 25 and 45, with complete osseous healing recorded by day 90. Notably, there was no swelling observed, and by day 23, the bone healing was reported as excellent [6]. Moawad et al. noted that the laser treatment group revealed significant alterations across most maxillofacial components following the expansion phase, although differences in retention or overall treatment duration were not found to be significant [14]. Angeletti et al. validated that the bone regeneration associated with laser use after SARPE exhibited a statistically significant difference. The laser group showed a higher mineralization rate (26.3%, *P *< 0.001) when compared to the control cohort [15].

Limitations and contraindications

During clinical trials, specific limitations were noted. In the research by Caccianiga et al., the issues arose from the analyzed sample size; however, the uniformity of the sample regarding age and gender distribution lessened this concern, even though there is a lack of clinical evidence in the current literature about differences in pain response and sensitivity between genders. Another drawback of this study is the subjective nature of patients’ self-reported pain evaluations and the absence of a placebo group, which would have enabled an assessment of how patient expectations affect pain perception [2]. The research conducted by Caccianiga et al. faced a similar limitation, making it crucial to note that the findings were based on a restricted sample size [9].

Matos et al. recognized the difficulties in selecting individuals with particular traits of posterior crossbite; however, it was essential to ensure a uniform pattern of transversal maxillary hypoplasia. To achieve the required subject numbers for the sample size calculation, a significant number of candidates had to be evaluated. Another constraint was accurately identifying the precise sites for laser treatment, given that the anatomical features of the palate differ among individuals. On the other hand, the laser's influence goes beyond its main target, affecting a wider suture area among patients. Furthermore, the existence of multiple laser devices with different specifications limited the capacity to compare and generalize the results [10].

Cepera et al. encountered difficulties in standardizing the retrieval process for radiographs, leading to some images being overlooked during the evaluation phase. This issue stemmed from complications within the existing radiograph protocol, which relied on the standardization of techniques, a single operator's presence, and patient availability at the university's radiology service [11]. In the study conducted by Abreu et al., regarding conventional rapid maxillary expansion for adults, its effectiveness was found to be limited by age, prompting the choice of SARPE instead [6]. Angeletti et al. pointed out that variability in palatine suture closure and differing bone regeneration following MPAS were limitations to consider, particularly in light of the preoperative constraints even in patients undergoing laser therapy [15].

The systematic review of clinical studies revealed no particular contraindications for applying laser or PBM in patients receiving RME or SARPE, apart from the finding by Abreu et al. that dental compensation should be avoided because of the risk of periodontal harm (gingival recession and root exposure) [6].

Santana et al. encountered limitations due to the inability to conduct a quantitative analysis, largely stemming from the considerable heterogeneity among the studies included. Key factors such as the survey methods, units of measurement, the stage of growth when treatment commenced, post-treatment follow-up protocols, and the application methods of the interventions differed significantly, contributing to this heterogeneity. Additionally, qualitative reviews have inherent limitations compared to mathematical synthesis, making it difficult to assess the data from individual studies accurately. Moreover, various adjunctive interventions were examined, but some methods had only a solitary study available for analysis. This situation influenced the certainty of evidence for several outcomes [17].

Outcomes

In their conclusions on clinical trials, Caccianiga et al. noted that LED PBM using the ATP38 laser reduced both the intensity and duration of pain experienced by a small group of patients undergoing RME [2]. Garcia et al. indicated that LLLT appears to facilitate the healing process during the retention phase post-RME [8]. Caccianiga et al. also found that orthodontic maxillary expansion, combined with unilateral corticotomy and LLLT, effectively resolved true unilateral crossbite [9]. Conversely, Matos et al. reported that PBM therapy (PBMT) did not accelerate bone regeneration in the MPS and that the levels of pain were similar [10]. Additionally, the research by de Rezende et al. indicated that LLLT with a GaAlAs diode (780 nm) did not affect postoperative mouth opening after SARPE [7]. Cepera et al. found that LLLT, when combined with RME, significantly aided in the opening of the MPS and enhanced the bone regeneration process, leading to faster healing [11]. Ferreira et al. discovered that LLLT had a favorable impact on bone regeneration at the MPS by accelerating the repair process [12]. Abreu et al. showed that laser therapy improves bone regeneration at the median palatal suture in patients who have undergone surgical-assisted rapid palatal expansion (SARPE) [6]. Additionally, da Fonseca et al. revealed in their research that PBM positively influences postoperative complications in various oral surgeries and supports bone repair after maxillary disjunction, whether assisted by surgery or not. They highlighted the necessity of evaluating its effects on pain, swelling, and numbness following surgery [13]. Moawad et al. demonstrated that laser-assisted rapid maxillary expansion (LARME) can significantly improve skeletal results in young adults with maxillary transverse deficiency (MTD) [14]. Angeletti et al. found that LLLT (GaAlAs) promotes bone healing in modified palatal expansion surgery (MPAS) after surgically assisted rapid maxillary expansion (SARME). However, after a seven-month follow-up, OD readings were lower compared to those recorded before the procedure [15].

In their comprehensive analysis for this paper, Davoudi et al. observed that LLLT could potentially support RME, given its absence of side effects, affordability, and the short duration of treatment required [1]. Lai et al. concluded that the addition of LLLT to RME protocols has demonstrated beneficial results through cellular biostimulation, improving angiogenesis, and fostering bone regeneration in the MPS [4]. Skondra et al. highlighted that LLLT seems to be an effective approach for promoting rapid bone healing and regeneration after the expansion of the MPS [5]. The study conducted by Santinoni et al. suggested that using LLLT post-surgery for maxillofacial bony defects may improve bone density. Furthermore, LLLT seems to provide anti-inflammatory and pain-relieving effects, aiding in recovery and enhancing oral health-related quality of life. However, to draw more conclusive results, the protocols for LLLT application need to be standardized [16]. Additionally, Santana et al. observed that the combination of LLLT with maxillary expansion leads to more efficient suture opening and bone healing. Limited evidence also indicates that osteoperforations might improve the skeletal results of RME in patients who are not in their growth phase. Currently, there are no supplementary methods to alleviate the periodontal side effects linked with RME [17]. In their thorough examination of this study, Farzan et al. noted that LLLT appears to be a promising approach for enhancing bone formation and can be utilized in RPE to decrease retention duration. Nonetheless, additional research, particularly long-term, prospective randomized controlled trials with larger participant groups, is essential to produce reliable findings and identify the optimal clinically applicable method [18].

## Conclusions

In orthodontics, LLLT is commonly used to ease discomfort from orthodontic adjustments, enhance bone healing following RME, and facilitate tooth movement. On the other hand, HILT serves as an option for addressing soft tissue issues associated with orthodontic care. All studies conducted uniformly agree that laser-based techniques offer significant advantages over currently utilized and broadly practiced methods, representing a challenging yet promising therapeutic option. The primary benefits of these techniques are their anti-inflammatory properties and reduced pain. Furthermore, these methods aid in bone healing, enhance recovery processes, and promote angiogenesis. Additionally, laser-based methods are considered safe and effective since they present no complications or side effects. However, some research indicates that these techniques do not necessarily lead to faster bone regeneration and pain levels were found to be similar. While initial findings are encouraging regarding the immediate facilitation of bone healing and regeneration after the MPS expansion, further well-structured studies, including double-blind clinical trials, are essential to determine the most effective protocol and establish more definitive conclusions on this matter. 
